# SugarSketcher: Quick and Intuitive Online Glycan Drawing

**DOI:** 10.3390/molecules23123206

**Published:** 2018-12-05

**Authors:** Davide Alocci, Pavla Suchánková, Renaud Costa, Nicolas Hory, Julien Mariethoz, Radka Svobodová Vařeková, Philip Toukach, Frédérique Lisacek

**Affiliations:** 1 Proteome Informatics Group, SIB Swiss Institute of Bioinformatics, 1211 Geneva, Switzerland; davide.alocci@sib.swiss (D.A.); julien.mariethoz@sib.swiss (J.M.); 2 Computer Science Department, University of Geneva, 1211 Geneva, Switzerland; 3 CEITEC–Central European Institute of Technology, Masaryk University Brno, 625 00 Brno-Bohunice, Czech Republic; pavla.suchankova@mail.muni.cz (P.S.); radka.svobodova@ceitec.muni.cz (R.S.V.); 4 National Centre for Biomolecular Research, Faculty of Science, 625 00 Brno-Bohunice, Czech Republic; 5 Polytech Nice Sophia, Campus SophiaTech, 06903 Sophia-Antipolis, France; renaud.costa@etu.unice.fr (R.C.); horynicolas@yahoo.fr (N.H.); 6 Zelinsky Institute of Organic Chemistry, Russian Academy of Sciences, Laboratory of Carbohydrate Chemistry, 119991 Moscow, Russia; netbox@toukach.ru; 7 Section of Biology, University of Geneva, 1211 Geneva, Switzerland

**Keywords:** carbohydrate, 2D structure, software, SNFG notation

## Abstract

SugarSketcher is an intuitive and fast JavaScript interface module for online drawing of glycan structures in the popular Symbol Nomenclature for Glycans (SNFG) notation and exporting them to various commonly used formats encoding carbohydrate sequences (e.g., GlycoCT) or quality images (e.g., svg). It does not require a backend server or any specific browser plugins and can be integrated in any web glycoinformatics project. SugarSketcher allows drawing glycans both for glycobiologists and non-expert users. The “quick mode” allows a newcomer to build up a glycan structure having only a limited knowledge in carbohydrate chemistry. The “normal mode” integrates advanced options which enable glycobiologists to tailor complex carbohydrate structures. The source code is freely available on GitHub and glycoinformaticians are encouraged to participate in the development process while users are invited to test a prototype available on the ExPASY web-site and send feedback.

## 1. Introduction

It is generally admitted that drawing glycans using a chemical notation can be at least cumbersome, if not a challenge. This issue was addressed very early in glycochemistry [1], and several groups have proposed symbolic nomenclatures to ease the representation of complex carbohydrates. Although these representations have evolved from the original idea to several visualization schemes reviewed in [2], the most compelling ones consist of a series of geometrical shapes that symbolize monosaccharide units connected with lines specifying glycosidic linkages. A recent reappraisal of this representation finally rallied a wide community of glycoscientists who have settled on the usage of the Symbol Nomenclature for Glycans (SNFG) [3]. As a result of the dissemination of this symbolic notation, a variety of software applications for drawing glycans has been developed to fulfil mainly two distinct purposes. A drawing interface may be useful to query databases or to input structures for further analysis, modelling, or prediction of properties. Conversely, the extent of glycan encoding formats often requires the translation of commonly used formats into images. The intrinsic user-friendliness of the symbolic nomenclature not only meets the glycoscientists’ needs but also simplifies access to glycoscience for non-specialists.

Over the past decades, the variety of glycan representations used in chemistry, glyco-chemistry and glycobiology gave rise to a series of editing tools. We however, limit our coverage to those that meet three requirements: (1) web-based, (2) freely accessible, and (3) exporting structures to standard encoding formats [2]. KegDraw can be considered as the earliest standalone online graphical glycan editor though it was preceded by a basic tool integrated in GlycoSuiteDB for graphical queries in the IUPAC condensed format used in that database [4]. KegDraw was designed to perform a similarity search in the KEGG databases where the original CarbBank [5] is integrated [6]. It was a Java application that needed to be installed and could produce low-resolution images where text labels of monosaccharides were connected by lines. GlycanBuilder [7] is a more recent java applet developed during the EuroCarb project [8]. This tool provides an interface to assemble glycan structures using the graphic visualization scheme proposed by the Consortium of Functional Glycomics (CFG) and described in [9]. GlycanBuilder was upgraded to work in a web environment, but it needs to be installed and connected with a server. Moreover, recent security upgrades of all major browsers seriously challenged the usage of Java applets. This web-based implementation usually involves fairly long time-lags during processing despite a recent upgrade [10]. However, this drawback can be avoided as demonstrated with the glycan structure builder of the GlycoViewer platform [11] a web interface for drawing glycans that pioneered in terms of design, usability and speed. A subjective weak point of this tool is the drag-and-drop implementation that allows computer users to draw a glycan structure easily with the help of a mouse. However, this may become tiresome if many and large structures are drawn on a touch-based device such as a tablet or a smartphone. Also, monosaccharides are displayed in text format like KegDraw neglecting the advantage brought by a symbolic notation. GlycoViewer, like GlycanBuilder, is composed of a client interface and a server written in Ruby on Rails. The drag-and-drop feature is also used in Glycano, a software for drawing glycans entirely written in JavaScript (http://glycano.cs.uct.ac.za). Glycano is browser-independent and does not require a server. The interface uses SNFG symbols, though not in the proper color scheme and lacks the option of positioning of monosaccharides according to their linkage. It is designed for trained chemists and glycobiologists precluding access for non-experts. Polys [12] and DrawGlycan-SNFG [13] are the most recent published tools to draw glycan structures. Polys is integrated in the Glyco3D portal [14] to mainly serve as an input form for building 3D models. This is also the case of the (unpublished) carbohydrate builder (http://glycam.org/tools/molecular-dynamics/oligosaccharide-builder/build-glycan?id=1) of the GLYCAM-Web portal. DrawGlycan-SNFG is standalone and produces high quality and SNFG-compliant depiction of glycan structures but data input is limited to IUPAC linear encoding [15]. Users cannot draw a structure interactively, but only use a string encoding to generate images.

In summary, tools developed in the last decade, are either incompatible with SNFG or rely on complicated and/or slow interfaces or have other format or usability limitations. From a technical viewpoint, these tools require a continuous connection with a server to support consistent and fast drawing. From a scientific viewpoint, we believe glycan drawing should be democratized. With this in mind, we have developed SugarSketcher, an intuitive and fast interface to draw glycans online. This tool is entirely built in JavaScript and does not need a connection to any server. It is supported by the major browsers and is fully compatible with the SNFG nomenclature. The interface has been streamlined to accommodate expert and non-expert usage. In particular, a “quick mode” allows users with limited knowledge of glycans to build up a structure quickly while the “normal mode” offers a broader range of options regarding the structural features of complex carbohydrates. Its beta-version is currently implemented as another graphic interface for searching structures in CSDB, the Carbohydrate Structure Database [16]. The GlycoCT [17] export feature allows every software project or a database supporting GlycoCT to translate the SugarSketcher user input into the project native notation and to further process the constructed glycan sequences, including piping data to the search engine. Major cheminformatics standards (Simplified Molecular-Input Line-Entry System (SMILES), InChi) are also available for export provided the structure is fully defined (e.g., no undetermined linkage or configuration). Furthermore, the code is destined to be shared and hopefully improved by the community of glycoinformaticians. A prototype of SugarSketcher is currently included in the tool collection of Glycomics@ExPASy [18] as a standalone application. It can be accessed at https://glycoproteome.expasy.org/sugarsketcher while the code is available on GitHub at https://github.com/alodavide/sugarSketcher.

## 2. Results

SugarSketcher is divided in two main components: the core JavaScript library and the D3.js (https://d3js.org)-based interface. This division provides two main advantages: (1) the core library can be used standalone or integrated in other web applications that handle information about glycan structures; (2) the interface can be modified by collaborators without changing the underlying core library. In the “Materials and Methods” section we present how the two components have been built using JavaScript and a set of libraries rich in visualization components.

The interface of SugarSketcher has been designed to address the increasing popularity of glycans among scientists with only basic knowledge of carbohydrates. Without knowledge of chemistry behind each monosaccharide, average users can quickly draw glycan and derivative structures using the “quick mode”. In this case, the interface presents 12 monosaccharides most commonly found in mammalian glycans as a reflection of the bias in structural data production observed in [19] for example. This limited set of monosaccharides is used as LEGO© bricks to sequentially build a structure. Each time a new monosaccharide is added to a structure, the user needs to input only the anomericity of the linkage and the attachment position of the monosaccharide. To depict monosaccharides and glycans, SugarSketcher uses the SNFG icons.

**Figure 1:**
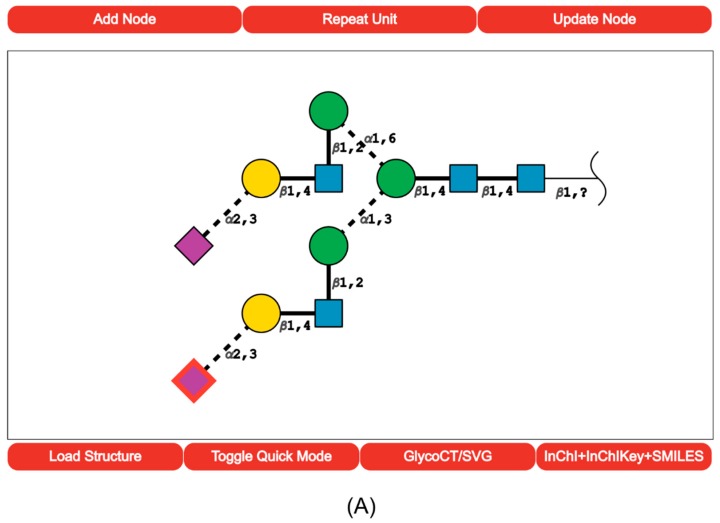

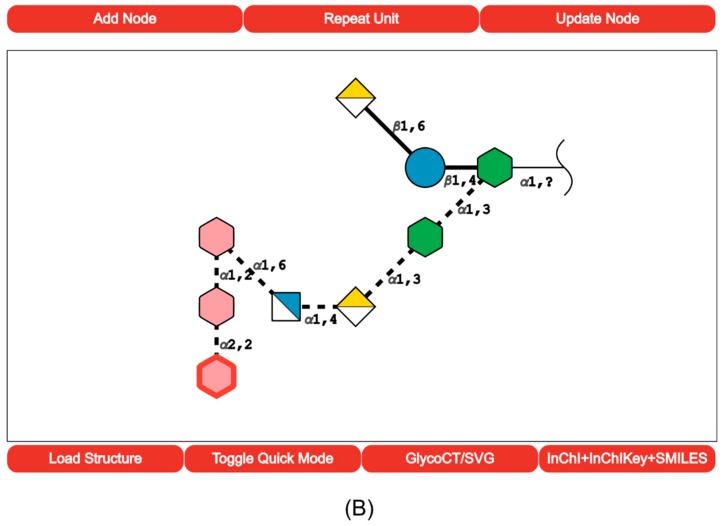
(**a**) Screenshot of the SugarSketcher interface after the completion of an N-glycan core carried out in the “quick mode”. The upper menu shows a selection of 12 monosaccharides more frequently observed in the composition of mammalian glycans. Monosaccharides are positioned following the option proposed in [16]. Linkage is indicated by the bond angle whereas anomericity by solid (β) or dashed (α) lines.; (**b**) Screenshot of the SugarSketcher interface after the completion of a glycan carried out in the “normal mode”. The top menu shows a much broader range of possible monosaccharides. The same positioning procedure applies.

Positioning monosaccharides basing on the acceptor linkage only can end up in overlays. For example, this situation occurs when two nodes coming from different branches should be in the same place. We have created a grid system that tracks whether a position is free or already occupied by a monosaccharide. At present, positioning follows the option of depicting the monosaccharide linkages with embedded type and anomericity [20]. Figure 1a shows a galactosylated and sialylated N-glycan core drawn with the “quick mode” option.

**Figure 2:**
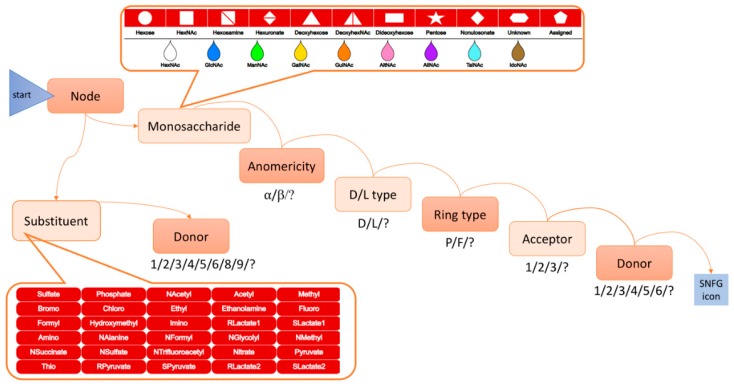
Drawing process of an SNFG icon in the “normal mode”. The first task is to select between a monosaccharide and a substituent. If the choice is “monosaccharide”, next, the user will select the geometrical and color attributes of a monosaccharide in the SNFG nomenclature from (1) an array of shapes and (2) an array of colors (framed at the top of this figure). If the choice is “substituent”, multiple buttons are displayed for selection (framed at the bottom of this figure). Then the user successively selects optional values (shown below each box) to specify further the characteristics of the ring and the linkage (anomericity, carbon acceptor and donor shown in boxes). This attribute selection process results in the placement of the corresponding monosaccharide/substituent on the screen. It is then repeated as many times as the targeted structure contains monosaccharides/substituents.

SugarSketcher provides a “normal mode” for glycochemists and glycobiologists. After switching off the “quick mode”, a user gains access to a more sophisticated menu. Now, the addition of a monosaccharide requires knowledge of stereochemistry and ring types, and not only acceptor, but also a donor linking position must be specified to create a glycosidic linkage. In the “normal mode”, the user can decorate monosaccharides with substituents and can add repeating units. In addition to the delete function available in the “quick mode”, an experienced user can modify each monosaccharide using the update button. Figure 1b shows an example of a bacterial LPS drawn in the “normal mode” following the drawing procedure summarized in Figure 2.

The overall drawing process is a succession of feature selection steps that ends with the display of an SNFG icon. This process is repeated as many times as the target structure contains building blocks. For the realization of Figure 1b example, the user is first invited to “Add Node” in the top menu. Mousing over this task reveals two options: monosaccharide and substituent. In the example the first entity is a monosaccharide (l-glycero-d-manno-heptose) which is therefore the selected option. Clicking on it prompts the display of a first array of geometrical shapes. In the example, clicking on the hexagon then prompts a second array of colors represented as drops. Clicking on the green drop will result in moving to the next step which is the sequential selection of options for anomericity, ring and linkage characteristics. Selecting optional values at each step (fully described in Supplementary S1–S5) will lead to the placement of the corresponding monosaccharide (alpha-linked l-glycero-d-manno-heptose) in main space of the interface.

SugarSketcher was benchmarked with currently available web interfaces in terms of speed for drawing a selection of small, middle-sized, and large molecules from several glycan databases. It was also compared to other tools according to a list of qualitative criteria. The results of the speed tests are summarized in S6 in Supplementary Material. Sugar Sketcher functionality and performance are compared to other tools in Table 1.

## 3. Discussion

We encourage glycoinformatics project holders to integrate SugarSketcher as an alternative structure input tool. Currently it has been incorporated in CSDB [16]. Ultimately it is also destined to become the graphic interface for querying databases and running software from the glycomics@ExPASy collection.

At the moment, SugarSketcher supports the depiction of glycan structures following the standard imposed by SNFG. However, there are various features that we would like to implement in the nearest future:

- On the fly Copy-Paste of the glycan image
- Import structure via the URL
- Automatic adjustment of parameters where it is possible to detect their chemically forbidden combinations

Several users have asked for the possibility of copying the glycan image from SugarSketcher to other applications via the clipboard. The choice of high-resolution Scalable Vector Graphics (SVG) as the primary image format precludes this possibility since no general-purpose software supports it. Other image formats will be included.

Another feature in development is the import of a glycan from the GlycoCT or IUPAC string in the URL parameter for an automatic image generation.

As SugarSketcher is developed primarily for non-glycobiologists, one of the main features we are working on is the possibility of automatic preselection of various parameters where it is possible to infer them from the user input. For example, the linking position in a donor residue (sometimes referred to as “anomeric carbon”) can be automatically detected based on the residue type (aldose vs. ketose). We provide in supplementary material, the non-exhaustive list of parameters/rules to be introduced in the next version to control the consistency of users’ input.

## 4. Materials and Methods

SugarSketcher is divided into two parts, the library and the interface. The library is a collection of JavaScript files which get compressed into a single file. The Interface is composed of nine files plus the index.html and two Cascading Style Sheet (CSS) files, which can be merged in one. In the end, the complete SugarSketcher needs 13 files.

### 4.1. The Core Library

The main assumption behind the core library is that each glycan can be represented as a graph. This concept has already been applied by users of MzJava [21] from which the core library is inspired. To avoid reinventing the wheel, the Graph class has been taken from Sigma.js, an established JavaScript library (http://sigmajs.org) which allows the integration of graphs in a web environment.

The Graph class, together with GraphNode and GraphEdge classes, form the general data structure that can handle a general graph. Since a glycan structure holds specific chemical information for each node and edge, the basic data structure is extended in the glycomics package to encapsulate glycan-specific information. For example, GraphNode is extended by Monosaccharide and Substituent classes. The Glycan class allows the creation of saccharide objects by connecting monosaccharides and substituents with glycosidic and substituent linkages, respectively.

**Figure 3:**
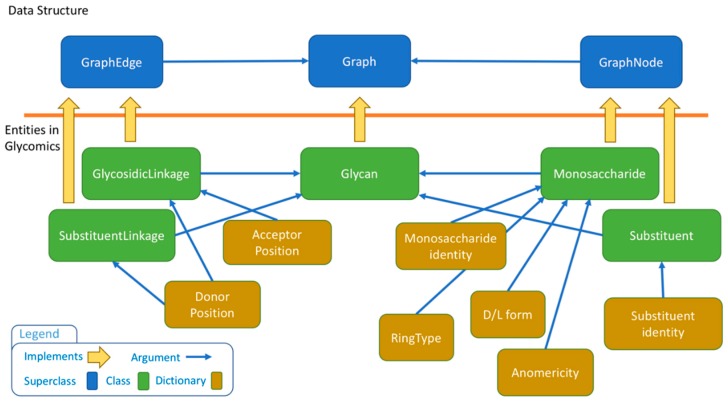
Entity relationships in the SugarSketcher data structure. The icon legend describes the color code for boxes.

To control data input, the core library provides a collection of dictionaries, one for each glycan-specific entity. These dictionaries encompass anomericity, ring type, isomer, etc. see Tables S1–S5. The user is required to pick an entry from the dictionary thereby blocking possible arbitrary inputs. At the level of nodes, the library provides dictionaries for MonosaccaridesTypes, SubstituentTypes, RingTypes and Anomericity. In addition, edges are defined from donor and acceptor linkage positions. The data structure is detailed in Figure 3 where the relationships between entities are detailed.

Since glycan structures are not always fully defined, the core library has been designed to handle fuzziness in both edges (linkage position) and nodes (monosaccharide type, its anomeric, absolute and ring size configurations). The user can input monosaccharides with multiple alternative connections on each carbon. Repeating parts of structure are handled by the repeating unit class and can be added to the glycan structure as single nodes. A collection of glycan structures is already encoded in the library and can be directly used to create sugar objects.

The input-output section of the library allows the import and export of glycan sequences. Since we are mainly using GlycoCT encoding [17] across the tools in Glycomics@ExPASy [18], the first implemented parser/writer implements the GlycoCT standard. Parsers and Writers are completely decoupled from the data structure allowing the implementation of adapters for any glycan encoding format. The way the library is built and the Apache license 2.0 (https://www.apache.org) allow any research group to contribute with their specific import and export adapters.

The core library has been designed to facilitate external contributions and encourage further extension. Unless the Graph class, the rest of the code follow the ECMAScript6 (ES6) standard and comes with unit test. The project includes resources for minification and transpilation to ECMAScript5 (ES5).

### 4.2. The Interface

SugarSketcher runs in two different modes: “quick mode” and “normal mode” the application of which is illustrated in the “results” section. In either mode, a collection of pre-built structures can be used as a template amenable to extension. This selection currently mirrors the trend in over-representation of animal N- and O-linked carbohydrate moieties in glycoproteins in recent databases and in the literature (except for CSDB [16]). All N-and O-linked core structures reported in [9] are listed. Additionally, a shortlist of glycan epitopes is provided. For example, to draw a di-antennary core-fucosylated N-linked structure, an N-linked core-fucosylated template can be loaded and the first antenna added manually. Then this antenna is selected and with the copy-paste functionality available on right click, the second antenna can be pasted to complete the structure. Mistakes can be corrected with the “Delete” button that has been enabled to prune the tree-like structure.

Once the structure is completed, the depiction can be downloaded in in high-resolution SVG format. As an alternative to images, SugarSketcher provides an export of glycan structures to the GlycoCT machine readable format [17]. Since several glycoinformatic tools provide the export in GlycoCT, SugarSketcher has an internal engine for parsing and displaying GlycoCT encoded structures. In that respect, GlycoCT encoded structures can also be imported and further modified in the SugarSketcher interface. We also added newly developed converters (unpublished) to extend export options to cheminformatics standards, namely SMILES, InChi (IUPAC International Chemical Identifier) and InChiKey, commonly adopted in major compound databases such as PubChem [22] and ChEBI [23]. At this stage, this export is restricted to fully defined structures, i.e., cannot be applied to structures with undetermined linkages or residues.

The interface is built using HTML5, CSS3 and the JavaScript library D3.js. (V3). To handle all glycan information, the interface is connected to the core library (see Section 4.1). SugarSketcher works with any browser that supports JavaScript (up to version ES6) and can be integrated in any website. In addition, it can be combined with web-based glycoinformatics tools, that accept GlycoCT as encoding format. For example, GlycoDigest [24] that simulates the digestion of glycans by exoglycosidases accepts the structure input in GlycoCT format. The same is true for several modern glycan databases, such as GlyTouCan [25] or CSDB [16].

## 5. Conclusions

To conclude, we are aware that SugarSketcher still shows weaknesses that are listed and further documented in the Supplementary Materials file. Nonetheless, we are actively attending to these items and invite users and developers to participate in the GitHub issue tracker to send feedback and report bugs.

## Figures and Tables

**Table 1:** Qualitative comparison of six tools generating SNFG-compatible glycan pictures.

	SugarSketcher [1]	POLYS Builder [2]	GlyTouCan [3]	CSDB Wizard [4]	GlycoViewer [5]	Carbohydrate Builder [6]
Library of pre-defined structures	yes	no	yes	yes	no	no
Edit a library; add substituents	yes	no	yes	via menu	yes	no
Selection of sugar residues via graphic symbols	yes	yes	yes	yes	no	no
Selection of sugar residues via text description	no	yes	no	yes	yes	yes
Clicks for a disaccharide *	16/6	8	12	9	7	10
Model time of a disaccharide * [min]	0:21/0:10	0:16	0:41	0:32	0:56	0:21
Import	GlycoCT, Library	INP (internal format)	GlycoCT, Library, CarbBank, Linucs, IUPAC, WURCS	GlycoCT, Library	no	no
Export	GlycoCT, SMILES, InChi, InChiKey, SVG	INP, PDB	GlycoCT, Glyde, Linucs, WURCS	GlycoCT, WURCS, SMILES, GLYDE-II, GLYCAM, LINUCS, MOL-file	no	PDB
Implementation	JavaScript	PHP, C	Java	PHP, JavaScript	Ruby, JavaScript	unknown

* For SugarSketcher, the first value is running in normal mode and the second in the Quick mode. Corresponding URLs: [1] https://glycoproteome.expasy.org/sugarsketcher/; [2] http://glycan-builder.cermav.cnrs.fr/; [3] https://glytoucan.org/Structures/graphical; [4] http://csdb.glycoscience.ru/database/core/wizard.html; [5] http://www.glycoviewer.babs.unsw.edu.au/sequence_sets/add.xhtml; [6] http://glycam.org/tools/molecular-dynamics/oligosaccharide-builder/build-glycan?id=1.

## Supplementary Materials

The following are available online at http://www.mdpi.com/1420-3049/23/12/3206/s1. (1) Installation; (2) List of missing features and shortcomings; (3) Known bugs; (4) Currently imposed constraints; (5) Open-to-discussion rules that would prevent user’s mistakes (to be implemented).

## Appendix A

The appendix contains details of the current procedure for importing the software, the full description of dictionaries in tables, lists of shortcomings and known bugs as well as suggestions for introducing consistency rules. All is saved in a Supplementary file.
