# Supplementary material for: SugarSketcher: Quick and Intuitive Online Glycan Drawing
Source: Molecules. 2018 Dec 5;23(12):3206. doi: 10.3390/molecules23123206 (PMC6320881; doi:10.3390/molecules23123206)
Supplement: Supplementary file 1 [file molecules-23-03206-s001.pdf]

1) Installation:

Current procedure for importing SugarSketcher in a website:
The first step to include SugarSketcher in a website is to check the library dependency. In order to work correctly, this software uses D3.js V3, d3.superformula, lodash and jQuery. If these libraries are not already imported, it is necessary to include them into the head of the target page, changing the source path according to where the files are placed.

```
<!—Library files-->
<script type="text/javascript" src="https://d3js.org/d3.v3.min.js"></script>
<script type="text/javascript" src="../js/views/d3.superformula.min.js"></script>
<script type="text/javascript" src="../js/views/jquery.min.js"></script>
<script type="text/javascript" src="../js/views/lodash.js"></script>
```

The second step involved the import of SugarSketcher files. These files can be copied from the application repository and added into the website project. As for the library, the user needs to import these files into the head of the target page coping the code below. Each file source must be changed according to the file position.

```
<!—SugarSketcher files-->
<link rel="stylesheet" href="../css/app.css">
<link rel="stylesheet" href="../css/menu.css">
<script src="../js/sb.js"></script>
<script src="../js/views/eventManager.js"></script>
<script src="../js/views/menu.js"></script>
<script src="../js/views/visualization.js"></script>
<script src="../js/views/chart.js"></script>
```

Once files are correctly imported, the HTML code in the body of the index.html page must be placed in the target page. Only, the div footer should not be copied.

To conclude the procedure, the line

```
<script src="app.js"></script>
```

need to be placed after the code. The source field should be completed with the correct path. You can see the index.html page as an example.

The future procedure would allow importing SugarSketcher editor by adding a single javascript line in any website. We have not investigated this procedure, but many other javascript web libraries are providing this possibility. So, we are confident that we can refactor SugarSketcher to improve the import procedure.

Current procedure for exporting in GlycoCT:
To export into GlycoCT, it is necessary to create an exporter.

1. var writer = new sb.GlycoCTWriter(glycan, treeData);
2. writer.exportGlycoCT();

TreeData and Glycan are defined by SugarSketcher

Dictionaries:

Table S1. List of Anomericities available in SugarSketcher.

Anomericity
Alpha
Beta
Undefined

Table S2. List of Enantiomer available in SugarSketcher.

D/L form
L
D
Undefined

Table S3. List of Ring Types available in SugarSketcher.

Ring Type
Pyranose
Furanose
Open
Undefined

Table S4. List of Substituents available in SugarSketcher.

Substituents
Acetyl
Bromo
Chloro
Ethyl
Ethanolamine
Fluoro
Formyl
Imino
Hydroxymethyl
R-Lactate-1
S-Lactate-1
Amino
Methyl
N-Acetyl
N-Alanine
N-Formyl
N-Glycolyl
N-Methyl
N-Succinate
N-Sulfate
N-Trifluoroacetyl
Nitrate
Phosphate
Pyruvate

Sulfate
Thio
R-Pyruvate
S-Pyryvate
S-Lactate-2
R-Lactate-2

Table S5. SugarSketcher supports all monosaccharides described in Table 1 of Ref 3. of the main text. This table shows monosaccharides available in the "Quick mode".

**Monosaccharide**
Galactose
Glucose
Mannose
Fucose
Xylose
N-Acetylglucosamine
N-Acetylgalactosamine
N-Acetylneuraminic acid
N-Glycolylneuraminic acid
Neuraminic acid
Ketodeoxynononic acid

2) List of missing features and shortcomings:

- Parameters like button colors, size of window, default zoom level etc. are hardcoded in the software as opposed to being isolated as a block for easy modification
- When a substituent is attached, its linking position is not displayed (e.g. Glc3OAc and Glc4OAc are shown the same way)
- Substitution positions at ring closure are not blocked (e.g. position 5 in aldofuranoses)
- Deoxygenated positions are not blocked (e.g. position 6 in Rhamnose)
- Cannot add phosphate as a residue in a backbone
- Cannot create two bonds between the same two residues (e.g. Gal4,6Py; or bonding of open form sugars; or biphosphates)
- No ringsize "linear"
- No way to enter alditols
- No aminoacids
- No fatty acids
- No inositols
- No more than 6 positions in the interface

3) Known bugs:

- Possibly dysfunctional:
    - copy-paste
    - repeating units
- reducing end
- possible unwanted superimposition of shapes and information about linkages
- impossible to avoid superimposition when new linkage is on the same carbon as a substituent

4) Currently imposed constraints:

- structure should start with a monosaccharide
- the substituent is placed around the monosaccharide according to the donor position (the position change if you select 3 or 6 carbon), but the position is not present as text.

5) Open-to-discussion rules that would prevent user's mistakes (to be implemented):

- anomeric position cannot be substituted (it can only substitute or not substitute another residue)
- acceptor position is 1 for aldoses, 2 for ketoses, and 3 for 3-uloses
- position of outgoing linkage of non-carbohydrate substituents is 1
- uronic acid cannot be substituted by O-linked sugars at its greatest position
- onic and ulosonic acids cannot be substituted by O-linked sugars at C1, however a nitrogen-bearing position can be linked to carboxy-bearing position in another residue
- any position beyond carbon skeleton (e.g. C6 in pentoses) cannot be substituted
- nitrogen-bearing position (e.g. C2 in GlcN) cannot be substituted by O-linked sugars
- deoxy-position (e.g. C6 in Fuc) cannot be substituted
- a position bearing a substituent (including acetyl group site in N-acetylated aminosugars) cannot be substituted
- ring closure (5 in aldopyranoses and in ketofuranoses, 6 in ketofuranoses, 4 in aldofuranoses) cannot be substituted
- any position cannot be substituted more than once, except if a substituent is in open form, or it is a phosphate, or a pyruvate.

Table S6: Test results of SugarSketcher for selected sugar molecules: a) Standard test cases and b) Challenging test cases.

a)

**Database**	**ID**	**Residue count**	**Creating time [min]**	**#Clicks**	**Quick mode [min]**	**Quick mode clicks**	**Most efficient [min]**	**Clicks**	**Notes**
Monosaccharide DB	8	1	0:05	8	0:01	1	0:01	1	Widespread
GlyTouCan	G86389LM	4	1:50	28	0:35	12	0:15	8	Anonymous linkage
CSDB	29745	4	1:12	32	-	-	0:56	22	Branched antigen + rare sugar
GlyTouCan	G97463WM	4	1:24	28	0:40	12	0:39	12	Anonymous anomericity
PubChem CID	91845634	8	1:36	28	1:25	12	0:20	14	Correct sugar
PubChem CID	91846062	8	3:02	56	1:05	24	0:24	14	Correct sugar
Glyconnect	3340	8	2:45	56	1:11	24	0:34	19	Correct sugar
UniCarbKB	5132	8	2:54	56	1:35	24	1:08	26	Anonymous linkage
GlyTouCan	G78526LZ	12	4:40	84	1:30	36	0:52	24	Correct sugar
GlyConnect	2021	12	4:20	84	1:40	36	0:46	28	Anonymous linkage/anomericity
Glycoscience.de	24967	12	4:35	84	1:33	36	0:40	24	Correct sugar

b)

**Glycan name**	**Residue count**	**Creating time [min]**	**#Clicks**	**Quick mode [min]**	**Quick mode clicks**	**Most efficient [min]**	**Clicks**	**Notes**
?DMan?(1-?)aLRhap	2	0:41	16	-	-	0:41	16	Unknown items
aDGlcf(1-2)bDGlcpN	2	0:52	16	-	-	0:33	11	Rare configuration
aDGlcp(1-2)HEX	2	0:44	16	-	-	0:30	11	Superclass
ECA trisaccharide	3	1:25	26	-	-	0:49	23	
Lewis X	3	0:49	24	0:1 3	9	0:02	2	
